# Intake, Growth and Carcass Traits of Steers Offered Grass Silage and Concentrates Based on Contrasting Cereal Grain Types Supplemented with Field Beans, Peas or Maize By-Products

**DOI:** 10.3390/ani13071209

**Published:** 2023-03-30

**Authors:** Rian Kennedy, Aidan P. Moloney, Edward G. O’Riordan, Alan K. Kelly, Mark McGee

**Affiliations:** 1 Teagasc, Animal & Grassland Research and Innovation Centre, Grange, Dunsany, Co. Meath C15 PW93, Ireland; 2 School of Agriculture and Food Science, University College Dublin, Belfield, Dublin 4 D04 V1W8, Ireland

**Keywords:** beef cattle, protein supplementation, nitrogen balance, native grains, oats, legume grains

## Abstract

**Simple Summary:**

In Europe, there is increasing interest in exploiting alternative indigenous energy and protein feedstuffs to increase self-sufficiency and sustainability. There is relatively little research information on oats or, field beans or peas as feed ingredients for beef cattle, particularly when used as supplements to grass silage. In terms of reducing nitrogen excretion and feed costs, it is also important to assess if protein supplements can be excluded from concentrate rations without compromising animal performance. This study found that the feeding value of rolled barley was similar to rolled oats and maize meal, and flaked beans and peas were similar to maize gluten feed and maize dried distillers grains when included in the supplementary concentrate to beef cattle offered grass silage. Excluding protein ingredients from a cereal-based concentrate did not affect animal performance and reduced nitrogen excretion.

**Abstract:**

The study objective was to determine intake and performance of beef cattle individually offered perennial ryegrass-dominant grass silage ad libitum supplemented with 4 kg dry matter daily of, rolled barley or maize meal-based concentrate rations containing supplements of flaked field beans, flaked peas, maize dried distillers grains (MDD) or maize gluten feed (MGF) for 110 days (Experiment 1), rolled barley or rolled oats with or without supplements of flaked field beans or flaked peas for 146 days (Experiment 2), and to quantify the nitrogen balance of diets similar to those offered in Experiment 2 (Experiment 3). The protein supplements were formulated to have similar crude protein concentrations. Cereal type or protein source did not affect intake, growth, feed efficiency and carcass traits in Experiment 1 or 2. Inclusion of a legume protein supplement with barley or oats had no effect on intake or growth performance (Experiment 2), whereas their exclusion decreased nitrogen intake, plasma urea concentrations and urinary and total nitrogen excretion (Experiment 3). The feeding value of barley was similar to oats and maize meal, and flaked beans and peas were similar to MGF and MDD, as supplements to grass silage. Excluding protein ingredients from a cereal-based concentrate did not affect animal performance and reduced nitrogen excretion.

## 1. Introduction

In temperate climatic regions, such as North Western Europe, the basal diet of beef cattle fed indoors is predominantly grass silage [1,2]. As silage alone usually does not supply sufficient nutrients to sustain target growth rates to reach a commercially acceptable carcass, it is supplemented with concentrates [1,3]. Concentrates fed to beef cattle are commonly comprised of a starch-rich cereal, complemented with a protein-rich feed ingredient to satisfy published protein requirements [4,5]. In Ireland, barley (*Hordeum vulgare*) and wheat (*Triticum*) predominate as cereal crops, and are widely used as concentrate supplements in the diet of beef cattle [4]. However Ireland, like many European countries, is a deficit animal feed country with a self-sufficiency in concentrate feeds of only 36% [6]. Imported feed ingredients, such as maize grain (*Zea mays*) and its by-products maize dried distillers (MDD) grains and maize gluten feed (MGF) [6], are used extensively in beef cattle concentrate rations [7,8,9]. There is increasing interest in further exploiting indigenous energy (e.g., alternative cereals such as oats (*Avena sativa*)) and ‘protein’ (e.g., grain legumes, faba beans (*Vicia faba*) and peas (*Pisum sativum*)) feedstuffs in Ireland and other European countries in order to reduce the over-reliance on imports [6,10]. Furthermore, the sustainability of ‘local’ arable farming can be improved by using oats and legume grains as ‘break’ crops within tillage rotations, by interrupting plant disease cycles and via nitrogen-fixing legumes lowering the requirement for inorganic fertiliser and improving soil fertility [11,12].

Faba beans and field peas, can be attractive alternative substitutes for ‘high-protein’ feed ingredients as well as cereals in ruminant diets because of their relatively high crude protein (CP) and starch concentrations [13,14]. Keller et al. [15] found that replacing maize grain and soyabean meal with faba beans in a concentrate supplement to a mixture of grass and maize silage for finishing bulls had no effect on intake, growth or carcass traits. Studies on the inclusion of peas in high-concentrate diets for finishing beef cattle replacing either dry-rolled maize plus rapeseed meal [16] or barley plus soya bean meal [17] reported no effects on intake, performance and carcass traits. Compared to dairy cows [18,19,20,21,22], research on the inclusion of beans and peas in concentrates offered as supplements to grass silage for beef cattle is sparse [14].

Maize grain is characterised by higher starch, and lower fibre and CP concentrations than barley [23]. The feeding value of maize grain can be influenced by the processing method used [24] such as very finely ground maize ‘meal’ [25], which is the predominant form of inclusion in concentrate beef rations in Ireland. Most research evaluating maize meal as a feed ingredient for finishing beef cattle typically entails high-concentrate diets and, when rolled barley is partially replaced by maize meal, animal intake and performance results are inconsistent across studies [26,27,28]. Oat grain is characterised by a higher fibre and fat concentration, a similar CP concentration, and a lower starch concentration than barley [23]. However, the limited research published evaluating its role as a feed ingredient for finishing beef cattle offered grass silage has found that replacement of rolled barley with rolled oats in the concentrate supplement decreased [29] or maintained [30] live weight gain and feed efficiency. This disparity in findings obtained across studies for cereal types requires elucidation, especially in the context of cereal-based concentrates supplemented with conventional ‘imported’ maize by-products, or locally-produced legume grains (i.e., field beans or peas) as ‘energy-protein’ sources for beef cattle offered grass silage.

Nitrogen excretion is a contributor to emissions of ammonia, nitrogen oxide and nitrate, which have detrimental environmental effects vis-à-vis air and water quality and greenhouse gas production [31]. Supplying protein above the ‘requirement’ of beef cattle results in excessive nitrogen excretion, mainly via urine [32,33]. A key strategy to reduce urinary nitrogen losses is to decrease the concentration of dietary CP [34,35]. In the context of increasing environmental regulations and the relatively high cost of protein feed sources, there is a need to improve nitrogen efficiency in beef production [14,36]. Although there is evidence to suggest that, overall, growth response to protein supplementation in finishing cattle offered grass silage-based diets is small [37], effects are inconsistent across feeding experiments. Responses to protein supplementation, in addition to that contained in barley, are generally obtained where silage digestibility and/or protein concentration is low, and where animal growth (protein deposition) potential is high [1], such as with ‘modern’ late-maturing suckler-bred cattle genotypes that currently predominate in Ireland. Consequently, it is important to assess and confirm if protein supplements can be excluded from cereal-based concentrate rations offered with relatively high-digestibility grass silage containing a moderately-high crude protein concentration, to cattle with a high genetic propensity for lean meat deposition without compromising animal performance, and to quantify the impact of this and alternative protein sources on nitrogen excretion.

Therefore, the objectives of the experiments outlined here were to determine, intake, growth and carcass traits of steers offered grass silage, supplemented with barley- and maize-based rations containing beans, peas, MGF or MDD (Experiment 1), or supplemented with barley and oats with or without beans and peas (Experiment 2), and to ascertain the apparent digestibility and nitrogen excretion of diets similar to those offered in Experiment 2 (Experiment 3).

## 2. Materials and Methods

This study, carried out at Teagasc Grange, Animal & Grassland Research and Innovation Centre, was approved by the Teagasc Animal Ethics Committee (Project RMIS No. 0808: TAEC236-2019; TAEC2020-292) and licenced by the Irish Health Products Regulatory Association (license numbers AE19132/I230; AE19132/P107), in accordance with the Cruelty to Animals Act 1876 and European Communities (Amendment of Cruelty to Animals Act 1876) Regulation 2002 and 2005.

### 2.1. Animal Growth Studies

#### 2.1.1. Animals and Pre-Experimental Management

Two experiments were conducted using spring-born, late-maturing breed (mainly Charolais/Limousin crossbred) suckler steers purchased from commercial livestock marts around Ireland and assembled in Teagasc Grange. Following arrival, animals were treated for internal and external parasites, and vaccinated as a prophylactic measure against clostridial and respiratory diseases. Prior to commencing the experiments, animals were offered grass silage ad libitum and 2 kg of a barley-based concentrate per head daily.

#### 2.1.2. Experimental Design and Dietary Treatments

##### Experiment 1

Eighty steers with an initial live weight of 575 ± 21.3 kg (mean ± standard deviation) were weighed on consecutive days, blocked on sire breed and mean live weight, and from within block were assigned at random to one of eight concentrate treatments in a 2 (rolled barley or maize meal) × 4 (supplements of flaked peas, flaked field beans, maize dried distillers plus solubles (MDD) grains or dried maize gluten feed (MGF)) factorial arrangement (Table 1 and Table 2). The duration of the experiment was 110 days. Concentrates were prepared as coarse rations and were formulated to be isonitrogenous based on analysis of samples of the primary individual feed ingredients obtained prior to the start of the experiment (Table 1). As the ruminal protein degradability of peas and beans is relatively high compared to MDD and MGF [38], peas and beans were flaked to decrease their degradability [39]. The flaking process entailed toasting with a 700 °C flame, bringing the product up to 135–140 °C for a dwell time of 9 min, followed by rolling and flaking while still hot and then cooling to ambient temperature.

##### Experiment 2

Seventy two steers with an initial live weight of 602 ± 22.0 kg were weighed on consecutive days, blocked on sire breed and mean live weight, and from within block, assigned at random to one of six concentrate treatments in a 2 (rolled barley or rolled oats only) × 3 (rolled barley or rolled oats plus supplements of flaked peas or flaked field beans) factorial arrangement (Table 1 and Table 3). The duration of the experiment was 146 days. Concentrates were prepared as coarse rations and concentrates containing peas or beans were formulated to be isonitrogenous.

#### 2.1.3. Animal Accommodation and Feeding

Animals were accommodated in a concrete slatted-floor building, grouped in pens of seven per treatment with pens within treatment equally distributed around the building. The lying area available in the pens was 2.85 m^2^/head. Animals were fed individually using Calan gates (American Calan, Northwood, NH, USA). All animals received grass silage ad libitum (0.05–0.10 in excess of the previous day’s intake) supplemented with 4 kg dry matter (DM) of their respective experimental concentrate supplement daily in two feeds. Fresh grass silage was offered each day and the weight of silage offered and refused was recorded daily; refusals were discarded twice weekly. The grass silage offered was prepared from a perennial ryegrass (*Lolium perenne*) dominant sward, cut using a conditioner mower and wilted for 24 h before harvesting using a precision-chop harvester. The silage was then compacted to expel air and sealed with two layers of polythene sheeting and weighted with tyres. Animals had continuous free access to clean, fresh water.

#### 2.1.4. Animal Measurements

Live weight was recorded on two consecutive days at the beginning and the end of each experiment and every 14 days throughout using a calibrated scales. Weighing was carried out in the morning, prior to feeding and daily live weight gain was calculated by linear regression regressing live weight on time for each animal. The depth of the *M. longissimus* muscle at the 3rd lumbar vertebra and the depth of subcutaneous fat at the 13th rib, the 3rd lumbar vertebra and the rump was measured at the beginning and end of each experiment using an automatic real-time scanner (model—ECM ExaGo Veterinary scanner, with a 3.5 MHz linear transducer, IMV imaging, Meath, Ireland), as described by Conroy et al. [40]. Gain in muscle and fat depth was calculated by the difference between the initial and final ultrasonic scans.

At the end of the experiments animals were transported 30 km to a commercial abattoir, and slaughtered within an hour of arrival according to EU standards. Cold carcass weight was estimated as 0.98 of the hot carcass and kill-out proportion was calculated as the cold carcass weight expressed as a proportion of pre-slaughter live weight. Carcasses were graded mechanically for conformation and fat score in accordance with the EU beef carcass classification scheme on a continuous 15-point scale [40]. To estimate carcass gain, the carcass proportion at the beginning of both experiments was assumed to be 0.53 of initial live weight [41].

### 2.2. Experiment 3—In Vivo Apparent Diet Digestibility, Rumen Fermentation and Nitrogen Balance

Twenty four spring-born, late-maturing suckler breed steers (417 ± 21.0 kg live weight; 11.7 ± 0.85 months of age) were blocked on live weight and randomly assigned to one of six concentrate treatments in a 2 (rolled barley or rolled oats only) × 3 (rolled barley or rolled oats plus supplements of flaked peas or flaked beans formulated to have similar crude protein concentrations) factorial arrangement (Table 4). 

Eight metabolism stalls were available, therefore the experiment comprised of 4 sequential phases, according to block, with 6 animals being used each phase. The experimental period was 24 days consisting of a 14-day dietary adaption period where steers were accommodated individually in a slatted floor shed, followed by a 10-day measurement period, where animals were accommodated individually in purpose-built metabolism stalls [42]. During the dietary adaption period, animals were individually offered grass silage ad libitum (proportionately 0.05–0.10 in excess of previous day’s intake) and the respective supplement at the same dietary proportion (0.60:0.40 grass silage and concentrates) as in Experiment 2. The concentrates were offered twice daily in two equal meals. Silage refusals were weighed daily to estimate DM intake and discarded. Following adaption, animals were moved to the metabolism stalls, and offered their respective diets at to 0.9 of ad libitum intake, to minimise between and within day variance in intake [42].

### 2.3. Sampling and Chemical Analysis

For Experiments 1 and 2, representative samples of the grass silage and concentrates offered were collected three times and twice weekly, respectively. Samples were stored at −20 °C prior to processing. Samples of silage and concentrates were pooled on a weekly basis for DM determination. Additional samples were composited on a 3-weekly basis for chemical analysis. Concentrate and silage DM was determined by oven drying 200 g samples at 90 °C for 16 h in a force draught oven. The grass silage DM concentration was corrected for loss of volatiles using an equation developed by Porter and Murray [43].

Determination of in vitro DM digestibility (DMD) and organic matter digestibility (OMD) of silages was carried out according to O’Kiely [44], and neutral cellulase plus gammanase digestibility (NCGD) of the concentrates was measured according to Lenehan et al. [5]. Neutral detergent fibre (NDF) and acid detergent fibre (ADF) concentrations were determined using the ANKOM Technologies filter bag technique [44]. Ash was determined using complete combustion in a muffle furnace for 5 h at 550 °C and CP concentration were measured using a Dumas-type N analyser (Leco FP-428; Leco Corporation, St. Joseph, MI, USA; CP = N × 6.25). Oil-B concentrations (acid hydrolysis/ether extract) were measured as described by Lenehan et al. [5]. Starch, water soluble carbohydrates, volatile fatty acids (VFA) and ammonia concentrations were measured using the methods described by O’Kiely [44]. The metabolisable protein (PDI: PDIN and PDIE) concentrations of the concentrates were estimated based on assigned values by INRAE-CIRAD-AFZ [38] for each individual feed ingredient, and the equations developed by Murphy et al. [45] (Table 2) were used to estimate the PDIN and PDIE concentrations of the grass silages.

For Experiment 3, feed sampling (daily) and intake, faeces, urine, blood and rumen sample collection, diet digestibility and nitrogen retention were determined as described by O’Connor et al. [42]. Samples were stored at −20 °C prior to processing. At the end of Experiment 3, urine and faecal samples for chemical composition were thawed and pooled on an equal weight basis, per steer, prior to laboratory analysis. Determination of feed chemical composition (as outlined above), faeces DM, N, ash and NDF concentrations, urine N concentration, rumen fluid pH and ammonia concentration, and plasma urea concentrations were carried out as described by O’Connor et al. [42].

### 2.4. Statistical Analysis

For all experiments, normality of residuals was checked using graphical methods: box-plot and scatter plot of residuals and fitted values, as part of the UNIVARIATE procedure of Statistical Analysis Software (SAS). Animal was the experimental unit. Data were statistically analysed using the general linear model procedure (PROC GLM) of SAS. The model contained fixed effects of block, cereal type, protein source and their interactions. Differences between treatment means were tested for significance using the PDIFF statement. Mean values were considered statistically significant when *p* < 0.05 and considered a tendency towards statistical significance when *p* < 0.10. For final ultrasonic measurements in Experiments 1 and 2, initial ultrasonic measurements were used as covariates.

## 3. Results

### 3.1. Experiment 1

Dry matter, chemical composition and in vitro digestibility of the primary individual concentrate feed ingredients, experimental concentrate rations and grass silage used in Experiment 1 are presented in Table 1 and Table 2, respectively. The grass silage offered had a moderately high DM concentration, high in vitro digestibility, a relatively low CP concentration (Table 2). Mean CP concentration of the concentrate rations was 141 g/kg DM. As the concentrates were only designed to have similar crude protein concentrations, the starch and fibre concentrations differed reflecting the inherent differences in the combined basal cereal and protein ingredients. The estimated UFL and PDI supplied by the peas, beans, MGF and MDD diets (including grass silage) were 0.99, 0.98, 0.97 and 0.98 UFL/kg DM and 81, 80, 83 and 82 g PDI/kg DM, respectively.

There were no interactions (*p* > 0.05) between cereal type and protein source for animal DM intake, performance or carcass traits (Table 5). Grass silage DM intake, total DM intake or DM intake scaled for live weight, daily live weight gain, feed conversion efficiency, slaughter weight, carcass weight, kill-out proportion, estimated carcass gain, and carcass conformation and fat scores were unaffected (*p* > 0.05) by cereal type or protein source.

Ultrasonic measures of mid-experiment (data not presented) and pre-slaughter fat depth and gain at the rib, lumbar and rump and *M. longissimus* muscle depth did not differ (*p* > 0.05) between the cereal types. However, rump fat gain and depth pre-slaughter was greater (*p* < 0.05) for beans and MGF compared to MDD with peas being intermediate (*p* > 0.05). All other ultrasonic measures of body composition did not differ (*p* > 0.05) between the protein sources.

### 3.2. Experiment 2

Dry matter, chemical composition and in vitro digestibility of the primary individual concentrate feed ingredients, experimental concentrate rations and grass silage used in Experiment 2 are presented in Table 1 and Table 3, respectively. The grass silage offered had a relatively low DM concentration, a moderately-high CP concentration and in vitro digestibility (Table 3). As intended, the CP concentration of the ‘cereal-only’ rations was lower (120 and 111 g/kg DM for barley and oats, respectively) than those supplemented with legumes, which had a mean CP concentration of 149 g/kg DM. The fibre and oil-B concentrations were higher and the starch concentrations were lower for the oats-based compared to the barley-based rations, reflecting the inherent differences in the basal cereals. The estimated UFL and PDI supplied by the ‘cereal’, peas and beans diets (including grass silage) were 0.92, 0.94 and 0.93 UFL/kg DM and 75, 82 and 82 g PDI/kg DM, respectively.

There were no interactions (*p* > 0.05) between cereal type and protein source for animal DM intake, performance or carcass traits (Table 6). Grass silage DM intake, total DM intake and total DM intake relative to live weight, daily live weight gain, feed conversion efficiency, slaughter weight, kill-out proportion, carcass weight, estimated carcass gain, carcass conformation score and carcass fat score did not differ (*p* > 0.05) between the cereal types or protein source, except for peas whereby there was a tendency (*p* = 0.08) for a reduction in daily live weight gain, feed conversion efficiency, slaughter weight, carcass weight and carcass conformation score compared to beans and ‘cereal’. Ultrasonic measures of pre-slaughter fat depth and gain at the rib, lumbar and rump and *M. longissimus* muscle depth did not differ (*p* > 0.05) for cereal type or protein source.

### 3.3. Experiment 3

Dry matter, chemical composition and in vitro digestibility of the experimental concentrate rations used in Experiment 3 are presented in Table 4. There were no interactions (*p* > 0.05) between cereal type and protein source for any of the intake, apparent digestibility, nitrogen balance, plasma urea, or rumen fermentation variables (Table 7).

Grass silage, concentrate and total DM or nitrogen intake, urinary and total nitrogen excretion, retained nitrogen, rumen pH and ammonia concentrations did not differ (*p* > 0.05) between the cereal types. The apparent digestibility of DM, organic matter and NDF (*p* < 0.01), and faecal nitrogen excretion (*p* < 0.05) was higher for barley compared to oats. Apparent nitrogen digestibility (*p* = 0.06) and plasma urea concentration (*p* = 0.07) tended to be higher for oats compared to barley. Protein source had no effect (*p* > 0.05) on intake, apparent digestibility, faecal nitrogen excretion, retained N, or rumen ammonia concentration. However, nitrogen intake (*p* < 0.01), urinary and total nitrogen excretion and plasma urea concentration (*p* < 0.05) were lower for cereal protein compared to peas and beans.

## 4. Discussion

Europe is deficient in high-protein animal feed ingredients, and it is now a declared policy goal of the European Commission and the member states to increase domestic production of plant-based protein and reduce dependency on imports [46]. Replacement of imported animal feed with traceable ‘locally-produced’ non-genetically modified feedstuffs, decreases the length of supply chains and also represents a significant opportunity for indigenous arable crop farmers. The overall purpose of this study was to evaluate the effect of utilising alternative ‘home-grown’ or ‘indigenous’ energy and energy-protein concentrate feed ingredients (i.e., oats, peas and beans) as supplements to grass silage for beef cattle in order to reduce imports and increase self-sufficiency in Ireland and other countries with similar temperate climates. Therefore, a primary objective in the formulation of the concentrate rations was to restrict the inclusion of feed ingredients to those produced in Ireland.

The proportion of concentrates in the dietary DM was approximately 0.40 in experiments 1 and 2, and the concentrates were fed ‘separately’ on the silage which, unlike TMR diets, permits the effect of supplement on silage intake to be quantified.

Animal DM intake relative to live weight in Experiments 1 (16.7 g/kg) and 2 (15.0 g/kg) was similar to previous studies where comparable suckler-bred genotypes were offered relatively high DMD (>715 g/kg) grass silage ad libitum and supplementary cereal-based concentrates at an equivalent ratio during the ‘finishing’ period (17.6 g/kg, McGee et al. [47]; 18.0 g/kg, Kelly et al. [8]; 15.1 g/kg, Doyle et al. [48]; 15.3 g/kg, Doyle et al. [3]). The corresponding mean daily live weight gain in Experiment 1 (0.98 kg) was also intermediate to the live weight gain range obtained in the previously cited studies (0.81–1.04 kg) and others (e.g., 1.08 kg, Regan et al. [49]; however, the mean growth performance obtained in Experiment 2, was less than (0.64 kg) typically found. Compared to Experiment 1, estimated dietary energy supply per kg DM was slightly lower (proportionately 0.05) for Experiment 2, and metabolisable protein (PDI) supply per kg DM was similar between both experiments. Although DM intake scaled for animal weight in Experiment 2 was at the lower end of expectations, there is no obvious explanation for the relatively low growth rate overall; however, because this occurred across all dietary treatments, it is unlikely that this influenced the relative differences obtained. As all animals underwent a clinical examination and were deemed healthy by a veterinary specialist, the overall ‘underperformance’ may be attributed to reduced compensatory growth potential possibly as a result of an excessively high plane of nutrition pre-purchase/finishing [50].

### 4.1. Cereal Type

Owens et al. [51] reviewed feeding trials of cattle fed high-concentrate diets, mainly in North America, and reported no significant difference in DM intake, average daily gain, or feed-to-gain ratio between barley or maize (corn), averaged across a range of processing methods, which concurs with the present findings. Likewise, Sutherland et al. [52] found no difference in DM intake, average daily gain or feed efficiency in ‘backgrounding’ steers offered barley silage or maize silage supplemented (0.45 of dietary DM) with either dry-rolled barley, dry-rolled maize or an equal mixture of both grains. In contrast, Steen [53] reporting on ‘unpublished’ research found that the feeding value of maize meal was 15% greater than rolled barley when offered to finishing beef cattle as a supplement to grass silage. The superior feeding value of maize meal was attributed to the relatively slower rate of ruminal fermentation of maize starch compared to barley starch, having a less negative effect on the ruminal digestion of the fibre in grass silage. However, finishing steers offered an isonitrogenous rolled barley plus soya bean meal-based ration or a maize meal plus maize gluten feed-based concentrate as a supplement to grass silage had similar intake and performance for both concentrate types [47]. In accord with Experiment 2, McGee et al. [30] also found that replacing rolled barley with rolled oats in a supplement to grass silage had no impact on intake, live weight gain, feed efficiency or carcass characteristics. In contrast, Huuskonen [29] observed lower live weight gain and poorer feed efficiency in dairy bulls offered a concentrate with increasing inclusion levels of oats as a supplement to grass silage. The oil concentration of the oats in the study by Huuskonen [29] was much higher than in the current experiment.

The chemical composition of the individual ingredients barley, maize meal and oats offered in the current experiments were within the ranges reported in feed databases [38,54]. Information in modern databases concerning feeding value indicates that barley is inferior (proportionately 0.86–0.95) to maize, and oats is inferior (proportionately 0.90–0.99) to barley [38,54], which is contrary to the results obtained in the current experiments. Steen [55] noted that the feeding value of feed ingredients as supplements to grass silage in practice did not reflect their published values, due to associative effects between the silage and concentrates affecting the nutrient utilisation of both the silage and the concentrates.

Collectively, the absence of a difference in Experiments 1 and 2 between the cereal types or an interaction between cereal and protein source for silage DM intake, animal live weight gain, feed efficiency, carcass weight and carcass traits, suggests that the feeding value of maize meal and rolled oats are similar to rolled barley as supplements to grass silage.

### 4.2. Protein Source

Where the concentrates were formulated to be isonitrogenous, this was achieved by solely changing the inclusion levels of the respective basal cereals and the ‘protein-energy’ ingredients. A consequence of this was that the quantity of each protein ingredient (and therefore cereal) included in the formulation differed between supplements, and they were purposefully not ‘balanced’ for other dietary components such as starch and fibre.

In terms of nutritive value, faba beans and peas have relatively high CP concentrations [38,54]; however, the degradability of faba bean and pea protein in the rumen is also very high—frequently in excess of 80%—compared to many other protein sources (e.g., 56%, maize distillers; 63%, soyabean meal) [14,38,39]. This suggests that peas and beans are not ideal protein supplements for grass silage [56], which is generally characterised by a low concentration of water soluble carbohydrates and a high proportion of soluble non-protein nitrogen [57,58,59]. To overcome this, the beans and peas in the current study were ‘flaked’ to decrease the relatively high rumen degradable protein [39,60]; this is a common industry practice.

The peas and faba beans used in experiments 1 and 2 had lower CP, higher NDF and ADF and similar starch concentrations as in published feed databases [38,54]. The chemical composition of the MGF was comparable, whereas MDD had higher ADF and lower NDF than published figures [38,54], which may be a result of differences in the primary manufacturing processes [61]. Peas and faba beans are assigned similar energy values in modern feed databases, whereas MDD and MGF are assigned relatively inferior feeding values of 0.97 and 0.92, respectively [38].

In beef cattle offered grass silage to appetite supplemented with concentrate, substitution of barley plus soyabean meal with MGF in the concentrate had no effect on silage intake, growth or carcass traits [8], whereas replacing barley plus soyabean meal with MDD in the concentrate had no effect on silage intake but increased live weight gain [7] or decreased silage intake without affecting growth performance [62]. The similar intake and performance for MGF and MDD in Experiment 2 is broadly consistent with these findings.

Beans did not affect intake or performance in either experiment, which is in line with the results of Keller et al. [15] who replaced SBM with beans in a supplement for finishing bulls fed maize plus grass silage and observed no significant differences in total DMI, growth, feed efficiency or carcass traits. Similarly, Cutrignelli et al. [63] replaced soyabean meal with faba beans in a supplement to a hay-based diet for finishing bulls reported no differences in overall growth rate between the protein sources. Compared to beans there were somewhat inconsistent results across Experiments 1 and 2 from including peas, with no difference in Experiment 1 and a tendency to negatively impact growth and feed efficiency in Experiment 2. There is no obvious reason for this disparity. In finishing beef cattle offered high-concentrate diets no difference in DM intake, live weight gain, feed efficiency or carcass traits were reported when peas replaced rolled maize plus rapeseed meal [16] or barley plus SBM [17] at increasing levels.

### 4.3. Protein Level

The mean total dietary (grass silage plus concentrates) CP supplied in Experiment 2 ranged from 133 g/kg DM for the cereal-only to 150 g/kg DM for the cereal plus protein supplement concentrates. This compares to 121 g/kg for Experiment 1, primarily reflecting the comparatively lower CP of the grass silage offered. Exclusion of supplemental protein from the concentrates in Experiment 2 did not adversely affect silage intake, daily live weight gain, feed efficiency or any carcass and carcass traits, which concurs with previous published literature on growing-finishing bulls offered high-digestibility grass silage [64,65] or a grass plus maize silage mixture [15]. This implies that cereals alone provide sufficient protein for growing-finishing cattle offered high nutritive value grass silage, which is 20–50% lower than many commercially-available concentrate rations. Similarly, from a review of the literature, McGee [1] concluded that in finishing steers offered grass silage, a performance response to protein supplementation in addition to that contained in barley was only likely when offered grass silage with low digestibility and/or low protein contents. From their meta-analysis, Huuskonen et al. [37] concluded that increasing dietary CP concentration increased live weight gain of growing-finishing cattle fed diets mainly based on grass silage or grass silage partly or completely replaced by whole-crop silages or straw, but the growth response was minimal, and that there was generally no benefit from protein supplementation when the grass silage-based diets were not limiting rumen undegradable protein supply.

Although formulating diets on the basis of CP is a widespread practice internationally, application of the metabolisable protein system is a more precise approach for reducing nitrogen intake and associated losses from beef cattle production [36].

The daily metabolisable protein (PDI) and net energy (UFL) requirements of a 630 kg late-maturing breed growing-finishing steer gaining 0.98 kg live weight per day (Experiment 1) are 770 g PDI and 8.1 UFL [66]. Corresponding values for a 650 kg steer gaining 0.64 kg live weight per day (Experiment 2) are 690 g PDI and 7.1 UFL. The mean dietary PDI supply in Experiment 1 for steers offered peas, beans, MGF and MDD (based on the lower of the two values for PDIE and PDIN), were 822, 835, 838 and 857 g PDI/day. Similarly, in Experiment 2 the mean dietary PDI supply for the ‘cereal’, peas and beans were 733, 798 and 797 g PDI/day. Correspondingly, the mean dietary UFL intake daily in Experiment 1 was 10.1, 10.2, 10.2 and 10.3 for peas, beans, MGF and MDD, and in Experiment 2 was 9.0, 9.1 and 9.0 for the cereal, peas and beans diets, respectively. In both experiments, the mean PDI and UFL supply exceeded the theoretical requirements. Based on these mean consumption values, animals in Experiment 1 had adequate PDI (and net energy) to sustain a daily growth rate well in excess of 1.0 kg. In Experiment 2, the cereal-only and protein-supplemented animals had sufficient PDI to sustain a daily growth rate of 0.77 and 1.0 kg, respectively, whereas all treatments had adequate net energy to sustain a growth rate in excess of 1.0 kg/day. Collectively, the inconsistency between dietary nutrient supply and animal performance based on the INRA protein (and energy) recommendations suggests that either the ‘supply’ is overestimated or the ‘requirements’ are underestimated for late-maturing breed steers offered grass silage-based diets. Similar conclusions were reached by Cantalapiedra-Hijar et al. [67] who compared two metabolisable protein levels to growing-fattening Charolais bulls offered a grass silage-based total mixed ration. Also, discrepancies in energy requirements of beef cattle across international feeding systems is recognised [68]. The INRA feeding system for beef cattle is established on a range of forage diets, especially maize silage rather than grass silage, and different feeding regimes and animal genotypes, which may partially explain the discrepancy.

### 4.4. Rumen Fermentation, Apparent Diet Digestibility and Nitrogen Balance

The lower rumen ammonia (numerically) and plasma urea concentrations for cereal compared to peas and beans, reflected the lower nitrogen intake and urinary excretion of nitrogen [42,69]. The lower apparent DM, organic matter and NDF digestibility for the oats-based diets compared to the barley-based diets concurs with the findings of Huuskonen [29], and can be attributed to oats-based diets containing higher NDF and ADF concentrations. Dry matter, organic matter, NDF and nitrogen digestibility were not influenced by protein source, which is consistent with the lack of relative differences in published digestibility figures [38], or by CP concentration implying that the total dietary CP concentration was providing sufficient rumen degradable protein to ensure high fibre digestibility [70].

The values for retained nitrogen for each treatment in the current study fell within the range of −6 to 109 g/d for steers fed grass silage-based diets [71]. On average, nitrogen use efficiency (NUE) of the treatments was close to the predicted 33% for beef steers fed grass silage-based diets [33]. Omitting a supplementary protein ingredient did not influence nitrogen retention, consistent with the similar growth performance between the comparable dietary treatments in Experiment 2, and resulted in (mainly) urinary excretion of the additional protein.

In the current experiment, reducing dietary CP level from 149 to 133 g/kg DM (11%) reduced total nitrogen excretion by 15.9%. Similarly, Keller et al. [70] reported a reduction in urine and total nitrogen excretion in Limousin bulls when protein ingredients were omitted from a cereal-based ration offered as a supplement to grass plus maize silage; in that study reducing dietary CP from 160 to 142 g/kg DM (11.3%) reduced total nitrogen excretion by 21%. Likewise, Kirwan et al. [72] using beef × dairy finishing heifers offered grass silage with a barley-based supplement found that reducing dietary CP from 164 to 133 g/kg DM (19%) reduced total nitrogen excretion by 20%. A reduction in nitrogen excretion, especially urinary, is critically important from an environmental perspective vis-à-vis ammonia and nitrous oxide emissions and nitrate leaching (Hristov et al., 2011).

Equations to predict nitrogen excretion from nitrogen intake by cattle offered grass silage-based diets [33,71] or a range of diets [35] have been developed. Applying the equations developed by Yan et al. [71], Jiao et al. [33] and Angelidis et al. [35] to our data overestimated total nitrogen excretion by 16, 1 and 6%, respectively. Application of the equations developed by Jiao et al. [33] and Angelidis et al. [35], which partitioned the nitrogen excretion into faecal and urinary nitrogen, to our data resulted in an underestimation of faecal nitrogen by 8% and 8%, and an overestimation of urinary nitrogen by 17 and 15%, respectively. Therefore, although the total nitrogen output can be predicted with a relatively close degree of accuracy, it is difficult to accurately predict separated faecal and urinary nitrogen excretion.

## 5. Conclusions

Under the conditions of these experiments, when included in the supplementary concentrate to grass silage, rolled oats had a similar feeding value to rolled barley, which had similar feeding value to maize meal for finishing beef cattle. Flaked beans and (possibly) peas are viable alternatives to MGF and MDD as protein-energy sources in concentrate supplements to grass silage, when required. The overall implication of this is that there can be a decreased dependence on imported feed ingredients. However, it should be noted that beef concentrate rations containing ‘by-products’, rather than human-edible feeds, substantially improve the human-edible protein ratio of grass-based beef production systems, which is extremely favourable from a ‘food-feed competition’ perspective [73].

Evidence is presented that where finishing steers are offered high-nutritive value grass silage, protein supplementation in addition to cereals (barley and oats) is not required vis-à-vis animal performance, which has feed cost-related benefits, as protein sources are usually more expensive. Furthermore, omitting additional protein reduced nitrogen excretion, which is beneficial for the environment.

## Figures and Tables

**Table 1:** Dry matter, chemical composition and in vitro digestibility of the individual primary feed ingredient used in Experiments 1 and 2.

	Experiment 1						Experiment 2			
	Rolled Barley	Maize Meal	Flaked Peas	Flaked Beans	Maize Gluten Feed	Maize Dried Distillers	Rolled Barley	Rolled Oats	Flaked Peas	Flaked Beans
Dry matter (g/kg)	798	869	840	813	890	900	829	877	909	834
Composition of dry matter (g/kg DM)										
Crude protein	113	91	223	275	221	318	122	111	226	281
Estimated PDIN ^1^	73	73	134	160	151	204	81	81	133	161
Estimated PDIE ^2^	87	84	156	190	102	154	101	84	156	190
Estimated UFL ^3^	1.11	1.29	1.21	1.21	1.11	1.17	1.11	1.00	1.21	1.21
Ash	22	14	31	35	72	63	22	25	30	33
Neutral detergent fibre	208	106	154	187	292	278	178	271	155	182
Acid detergent fibre	70	46	94	137	98	161	50	134	64	126
Starch	589	594	449	373	116	17	592	420	458	381
Oil-B	26	42	17	17	70	78	26	34	19	14
NCGD ^4^	874	915	956	929	755	758	864	662	912	923

^1^ PDIN: PDIA + PDIMN; ^2^ PDIE: PDIA + PDIME, INRAE-CIRAD-AFZ (2021); ^3^ UFL = Feed unit values from INRAE-CIRAD-AFZ (2021); ^4^ NCGD = Neutral cellulase gammanase digestibility.

**Table 2:** Dry matter, chemical composition and in vitro digestibility (g/kg DM unless otherwise stated) of the perennial ryegrass-dominant grass silage and experimental concentrate rations used in Experiment 1.

	B-P ^1^	B-B	B-MGF	B-MDD	M-P	M-B	M-MGF	M-MDD	Grass Silage
Rolled barley	620	729	624	776	-	-	-	-	-
Maize meal	-	-	-	-	522	645	524	706	-
Flaked peas	315	-	-	-	413	-	-	-	-
Flaked beans	-	206	-	-	-	291	-	-	-
Maize gluten feed	-	-	311	-	-	-	411	-	-
Maize distillers	-	-	-	159	-	-	-	229	-
Molasses	37	37	37	37	37	37	37	37	-
Minerals/vitamins	28	28	28	28	28	28	28	28	-
Composition of dry matter									
Dry matter (g/kg)	805	793	805	801	836	829	841	837	413
Crude protein	140	138	138	138	143	137	148	142	109
Estimated PDIN ^2^	88	87	93	89	93	94	100	98	71
Estimated PDIE ^2^	103	103	86	92	108	109	86	95	76
Ash	65	65	63	72	58	60	62	55	69
Neutral detergent fibre	124	140	182	161	96	97	200	138	502
Acid detergent fibre	60	78	69	63	61	61	71	63	293
Starch	528	511	421	442	521	511	393	478	-
Oil-B	23	23	31	33	26	29	43	45	-
NCGD ^3^	872	856	834	841	906	890	828	853	-
Dry matter digestibility	-	-	-	-	-	-	-	-	776
Organic matter digestibility (g/kg)	-	-	-	-	-	-	-	-	767
DOMD ^4^	-	-	-	-	-	-	-	-	714
pH	-	-	-	-	-	-	-	-	3.9

^1^ B-P = Barley + Peas, B-B = Barley + Beans, B-MGF = Barley + Maize gluten feed; B-MDD = Barley + Maize dried distillers’, M-P = Maize meal + Peas, M-B = Maize meal + Beans, M-MGF = Maize meal + Maize gluten feed; M-MDD = Maize meal + Maize dried distillers’; ^2^ PDIN and PDIE of concentrates calculated based on ingredient proportions in the ration, Silage PDIN: 6.84 + 0.602(CP) + 0.032 (Ash) − 0.005 (DMD), Silage PDIE: 27.7 + 0.083 (DMD) − 0.147 (CP); ^3^ NCGD = Neutral cellulase gammanase digestibility; ^4^ DOMD = Digestible organic matter in the DM.

**Table 3:** Ration composition (g/kg DM), dry matter, chemical composition and in vitro digestibility (g/kg DM unless otherwise stated) of the perennial ryegrass-dominant grass silage and experimental concentrate rations used in Experiment 2.

	B ^1^	B-P	B-B	O	O-P	O-B	Grass Silage
Rolled barley	935	654	752	-	-	-	-
Rolled oats	-	-	-	935	644	748	-
Flaked peas	-	281	-	-	291	-	-
Flaked beans	-	-	183	-	-	185	-
Molasses	37	37	37	37	37	37	-
Minerals/vitamins	28	28	28	28	28	28	-
Composition of dry matter							
Dry matter (g/kg)	803	814	800	828	840	829	253
Crude protein	120	147	153	111	144	151	149
Estimated PDIN	75	90	90	76	91	91	96
Estimated PDIE	94	110	111	78	99	98	67
Ash	58	59	57	56	61	59	95
Neutral detergent fibre	162	156	159	253	192	243	534
Acid detergent fibre	67	72	85	136	111	139	331
Starch	522	508	505	418	433	405	-
Oil-B	22	21	20	37	34	31	-
NCGD ^2^	855	857	848	742	805	780	-
Dry matter digestibility	-	-	-	-	-	-	745
Organic matter digestibility (g/kg)	-	-	-	-	-	-	734
DOMD ^3^	-	-	-	-	-	-	662
pH	-	-	-	-	-	-	4.2

^1^ B = Barley, B-P = Barley + Peas, B-B = Barley + Beans, O = Oats, O-P = Oats + Peas, O-B = Oats + Beans; ^2^ NCGD = Neutral cellulase gammanase digestibility; ^3^ DOMD = Digestible organic matter in the DM.

**Table 4:** Dry matter, chemical composition and in vitro digestibility (g/kg DM unless otherwise stated) of the perennial ryegrass-dominant grass silage and experimental concentrate rations used in Experiment 3.

	B ^1^	B-P	B-B	O	O-P	O-B	Grass Silage
Rolled barley	935	668	750	-	-	-	-
Rolled oats	-	-	-	935	650	743	-
Soybean meal	-	-	-	-	-	-	-
Flaked peas	-	267	-	-	285	-	-
Flaked beans	-	-	185	-	-	192	-
Molasses	37	37	37	37	37	37	-
Minerals/vitamins	28	28	28	28	28	28	-
Composition of dry matter							
Dry matter (g/kg)	844	843	842	845	844	843	222
Crude protein	114	152	152	108	152	147	147
Ash	54	54	53	43	48	44	106
Neutral detergent fibre	256	212	251	203	161	168	561
Acid detergent fibre	114	100	126	51	67	70	343
Starch	661	654	674	840	747	715	-
Oil-B	43	36	38	31	24	24	-
NCGD ^2^	821	909	891	953	969	942	-
Dry matter digestibility	-	-	-	-	-	-	716
Organic matter digestibility (g/kg)	-	-	-	-	-	-	696
DOMD ^3^	-	-	-	-	-	-	629
pH	-	-	-	-	-	-	3.9

^1^ B = Barley, B-P = Barley + Peas, B-B = Barley + Beans, O = Oats, O-P = Oats + Peas, O-B = Oats + Beans; ^2^ NCGD = Neutral cellulase gammanase digestibility; ^3^ DOMD = Digestible organic matter in the DM.

**Table 5:** Effects of cereal-based concentrate type and protein source on dry matter (DM) intake, growth, feed conversion efficiency (FCE), ultrasonic measures of body composition and carcass traits of steers in Experiment 1.

	Cereal Type (CT)			Protein Source (PS)					Significance ^2^	
	Barley	Maize	SEM ^1^	Peas	Beans	Gluten	Distillers	SEM	CT	PS
Silage DM intake (kg/day)	6.5	6.4	0.09	6.2	6.4	6.5	6.5	0.14	NS ^3^	NS
Total DM intake (kg/day)	10.5	10.4	0.09	10.2	10.4	10.5	10.5	0.14	NS	NS
DM intake/kg live weight (g)	16.8	16.7	0.13	16.4	16.7	16.9	16.9	0.20	NS	NS
Daily live weight gain (kg)	0.98	0.99	0.031	0.96	0.96	1.02	0.99	0.046	NS	NS
FCE (g live weight gain/kg DM intake)	93.0	95.0	2.61	93.9	91.4	97.3	93.3	3.91	NS	NS
Pre-slaughter ultrasound measurements (mm) ^4^										
Final rib fat depth	6.3	6.2	0.25	6.5	6.4	6.0	6.0	0.35	NS	NS
Final lumbar fat depth	4.1	4.1	0.17	4.0	4.4	3.8	4.3	0.24	NS	NS
Final rump fat depth	12.3	11.7	0.44	11.4 ^bc^	13.5 ^a^	12.8 ^ab^	10.4 ^c^	0.65	NS	<0.01
Final *M. longissimus* depth	77.8	76.9	0.70	77.5	76.3	78.1	77.7	1.09	NS	NS
Rib fat gain	3.7	3.5	0.24	3.9	3.7	3.4	3.4	0.35	NS	NS
Lumbar fat gain	2.3	2.3	0.17	2.2	2.6	2.0	2.5	0.24	NS	NS
Rump fat gain	8.6	8.0	0.44	7.7 ^bc^	9.7 ^a^	9.1 ^ab^	6.7 ^c^	0.64	NS	<0.01
*M. longissimus* gain	8.3	7.4	0.74	8.0	6.7	8.5	8.1	1.09	NS	NS
Slaughter weight (kg)	680	681	3.4	678	678	685	681	5.1	NS	NS
Carcass weight (kg)	388	385	2.4	390	383	387	384	3.6	NS	NS
Kill-out proportion (g/kg)	570	565	2.9	576	566	565	564	4.3	NS	NS
Estimated carcass gain (kg/day)	0.77	0.74	0.022	0.79	0.73	0.76	0.73	0.033	NS	NS
Carcass conformation score (1–15)	8.5	8.2	0.19	8.8	8.2	8.1	8.3	0.28	NS	NS
Carcass fat score (1–15)	6.4	6.9	0.23	6.4	6.8	7.0	6.3	0.34	NS	NS

^1^ SEM = Standard error of the mean; ^2^ No interactions were observed between cereal type and protein source; ^3^ NS: not significant; ^4^ Initial ultrasonic scan used as a covariate ^a–c^ LS means with different superscripts were significantly different.

**Table 6:** Effects of cereal-based concentrate type and protein source on dry matter (DM) intake, growth, feed conversion efficiency (FCE), ultrasonic measures of body composition and carcass traits of steers in Experiment 2.

	Cereal Type (CT)			Protein Source (PS)				Significance ^2^	
	Barley	Oats	SEM ^1^	‘Cereal’	Peas	Beans	SEM	CT	PS
Silage DM intake (kg/day)	5.8	5.6	0.11	5.8	5.7	5.7	0.14	NS ^3^	NS
Total DM intake (kg/day)	9.8	9.6	0.11	9.8	9.7	9.7	0.14	NS	NS
DM intake /kg live weight (g)	15.1	14.8	0.15	15.0	14.9	14.9	0.19	NS	NS
Daily live weight gain (kg)	0.64	0.64	0.028	0.68	0.58	0.67	0.035	NS	0.08
FCE (g live weight gain/kg DM intake)	65.5	66.6	2.59	69.3	59.9	68.8	3.29	NS	0.08
Pre-slaughter ultrasound measurements (mm) ^4^									
Final rib fat depth	5.6	5.7	0.23	5.5	5.6	5.9	0.30	NS	NS
Final lumbar fat depth	3.1	3.1	0.13	2.9	3.3	3.1	0.16	NS	NS
Final rump fat depth	6.1	6.5	0.37	6.4	5.9	6.5	0.47	NS	NS
Final *M. longissimus* depth	74.4	74.2	0.86	75.0	73.2	74.7	1.08	NS	NS
Rib fat gain	2.5	2.7	0.23	2.4	2.6	2.8	0.30	NS	NS
Lumbar fat gain	0.8	0.9	0.14	0.6	1.1	0.8	0.17	NS	NS
Rump fat gain	2.4	2.8	0.37	2.7	2.2	2.8	0.47	NS	NS
*M. longissimus* gain	3.0	2.7	0.86	3.6	1.7	3.3	1.06	NS	NS
Slaughter weight (kg)	697	697	4.0	703	688	701	5.0	NS	0.08
Carcass weight (kg)	402	405	3.3	410	396	405	4.2	NS	0.08
Kill-out proportion (g/kg)	577	581	3.3	583	576	578	4.2	NS	NS
Estimated carcass gain (kg/day)	0.57	0.58	0.023	0.62	0.52	0.59	0.029	NS	0.08
Carcass conformation score (1–15)	9.0	9.5	0.24	9.3	8.7	9.7	0.31	NS	0.08
Carcass fat score (1–15)	7.9	7.8	0.24	7.9	7.8	7.9	0.30	NS	NS

^1,2,3,4^ See footnotes Table 5.

**Table 7:** Effects of cereal-based concentrate type and protein source on dry matter (DM) intake, apparent digestibility, nitrogen (N) balance, plasma urea and rumen pH and ammonia concentrations of steers in Experiment 3.

	Cereal Type (CT)			Protein Source (PS)				Significance	
	Barley	Oats	SEM ^1^	‘Cereal’	Peas	Beans	SEM	CT ^2^	PS
Silage DM intake (kg/d)	4.3	4.1	0.12	4.1	4.3	4.3	0.15	NS ^3^	NS
Concentrate DM intake (kg/d)	2.8	2.6	0.07	2.6	2.7	2.8	0.09	NS	NS
Total DM intake (kg/d)	7.1	6.7	0.19	6.7	7.0	7.1	0.24	NS	NS
Digestibility									
Dry matter	744	720	5.7	733	730	734	7.2	<0.01	NS
Organic matter	762	736	5.4	746	751	750	6.8	<0.01	NS
Neutral detergent fibre	671	624	9.0	639	654	650	11.4	<0.01	NS
Nitrogen	635	664	10.4	636	647	665	13.3	0.06	NS
Nitrogen intake (g/d)	164	154	4.3	143 ^a^	167 ^b^	168 ^b^	5.5	NS	<0.01
Nitrogen loss									
Faecal (g/d)	60	52	2.7	52	59	56	3.4	<0.05	NS
Urinary (g/d)	51	52	2.5	43 ^a^	55 ^b^	56 ^b^	3.1	NS	<0.05
Total (g/d)	111	103	3.7	95 ^a^	114 ^b^	112 ^b^	4.7	NS	<0.05
Retained Nitrogen									
g/d	54	51	3.2	48	53	56	4.1	NS	NS
g/kg absorbed	473	486	17.8	476	466	496	22.4	NS	NS
g/kg live weight	120	113	6.5	107	118	125	8.2	NS	NS
g/kg nitrogen intake (NUE ^4^)	326	326	17.8	332	314	332	22.4	NS	NS
Rumen pH	6.8	6.7	0.06	6.7	6.8	6.7	0.07	NS	NS
Rumen ammonia (mg/L)	132	152	10.3	119	161	148	13.0	NS	NS
Plasma urea (mmol/L)	3.4	3.8	0.17	3.0 ^a^	3.8 ^b^	4.0 ^b^	0.21	0.07	<0.05

^1,2,3^ See footnotes Table 5; ^4^ NUE = Nitrogen use efficiency; ^a–b^ LS means with different letters were significantly different.

## Data Availability

The datasets used and analysed during the current study are available from the corresponding author on reasonable request.
